# Investigating rituximab-induced infusion-related reactions in patients with non-Hodgkin lymphoma, with a focus on follicular lymphoma: a retrospective observational study

**DOI:** 10.1186/s40780-026-00538-6

**Published:** 2026-01-12

**Authors:** Sachiko Hirobe, Koji Fukada, Mayu Miyamoto, Moeno Inukai, Rinka Fujimoto, Shinichiro Maeda, Makiko Maeda, Yasushi Fujio

**Affiliations:** 1 Laboratory of Clinical Pharmacology and Therapeutics, Graduate School of Pharmaceutical Sciences, The University of Osaka, 1-6 Yamadaoka, Suita, Osaka 565-0871 Japan; 2 Department of Pharmacy, The University of Osaka Hospital, 2-15 Yamadaoka, Suita, Osaka 565-0871 Japan; 3 Department of Molecular Pharmaceutical Science, Graduate School of Medicine, The University of Osaka, 2-2 Yamadaoka, Suita, Osaka 565-0871 Japan; 4 Laboratory of Clinical Science and Biomedicine, Graduate School of Pharmaceutical Sciences, The University of Osaka, 1-6 Yamadaoka, Suita, Osaka 565-0871 Japan; 5 Integrated Frontier Research for Medical Science Division, Institute for Open and Transdisciplinary Research Initiatives, The University of Osaka, 1-1 Yamadaoka, Suita, Osaka 565-0871 Japan; 6 Medical Center for Translational Research, Graduate School of Medcine, The University of Osaka, 2-2 Yamadaoka, Suita, Osaka 565-0871 Japan

**Keywords:** Infusion-related reaction, Rituximab, Lymphoma, Retrospective observational study, Cytokine, Chemokine

## Abstract

**Introduction:**

The incidence of infusion-related reactions (IRRs) caused by rituximab is very high, and many studies have explored factors related to the onset of IRR; however, most analyses have been conducted on a mixed range of disease indications. This study aimed to investigate rituximab-induced IRR in patients with non-Hodgkin lymphoma (NHL), particularly follicular lymphoma (FL), and examine the factors related to its occurrence.

**Methods:**

Data on sociodemographic and blood test findings were collected on the closest possible date prior to rituximab administration. Cytokine/chemokine levels in the blood samples were measured before the drug administration. During rituximab administration, vital signs, including pulse, oxygen saturation, respiratory rate, and body temperature, were carefully monitored. IRRs were defined as adverse reactions that occurred within 24 hours of drug administration, the grades of which were assessed using the Common Terminology Criteria for Adverse Events, version 5.0.

**Results:**

IRRs were reported in 119/252 NHL cases (47.2%), with incidences of 36/61 (59.0%) FL cases and 48/119 (40.3%) diffuse large B-cell lymphoma (DLBCL) cases; the incidence of IRR was significantly higher in FL (*p* = 0.0174). Logistic regression analysis in FL revealed a significant correlation between IRR expression and lymphocyte counts of ≥ 1,600/µL (odds ratio 14.0, *p* = 0.0162). Additionally, interferon-γ (*p* = 0.0262), chemokine ligand 26 (*p* = 0.0394), and vascular endothelial growth factor (*p* = 0.0359) levels in FL.

**Conclusions:**

The incidence of IRR differed significantly between patients with FL and those with DLBCL. In FL, rituximab is administered while monitoring the disease condition and when the tumor burden is relatively large, which is thought to increase the frequency of IRR, suggesting that lymphocyte count can lead to effective prediction. The occurrence of IRR can be predicted by measuring the blood cytokine and chemokine levels before rituximab treatment, leading to the provision of safe and useful treatment.

**Supplementary information:**

The online version contains supplementary material available at 10.1186/s40780-026-00538-6.

## Background

Rituximab (Rituxan^®^) is a mouse or human chimeric monoclonal antibody that binds to CD20 on the surface of human B lymphocytes. It was developed in the United States as a treatment for B-cell non-Hodgkin lymphoma (NHL) and approved in November 1997. In Japan, it was approved in June 2001 as a therapeutic agent for CD20-positive low-grade or follicular B-cell NHL and mantle cell lymphoma. It is also used to treat intractable nephrotic syndrome and CD20-positive chronic lymphocytic leukemia. Rituximab exhibits antitumor activity via a mechanism of action different from that of cytotoxic agents, such as complement-dependent cytotoxicity and antibody-dependent cellular cytotoxicity (ADCC). Characteristic adverse drug reactions such as infusion-related reactions (IRRs) have also been reported.

IRR is an adverse drug reaction associated with rituximab administration within 24 hours of administration. These symptoms are usually similar to hypersensitivity or allergic symptoms; however, serious adverse drug reactions, such as anaphylaxis-like symptoms, pulmonary damage, cardiac damage, and even death, have been reported. The IRR expression rate is high at the first administration and decreases with subsequent administration. Therefore, medical staff should monitor patients who receive the initial administration. The package insert recommends the use of antipyretic analgesics and antihistamines as premedications to prevent and suppress IRR.

Much research has been conducted on the prediction and suppression of IRR. The reduction in the IRR expression rate due to premedication of corticosteroids, high incidence rate in low-grade lymphoma, and correlation between lymphocyte count and IRR expression are previously reported [1, 2]. However, they include not only NHL, but also chronic lymphocytic leukemia and autoimmune diseases [2–5]. As clinical characteristics differ among the indications, it is necessary to narrow down the subject of clinical research to predict IRR with higher accuracy. Several studies have focused on cases of NHL, but included multiple lymphoma subtypes, and the comparison of IRR expression between these disease types has been insufficient [6–10]. In addition, there are some reports that do not limit the analysis to the first administration [11]. From the perspective of predicting the occurrence of IRR, it is important to analyze patient information at the time of the first administration, which has a high frequency of IRR. This study investigated the IRRs, while targeting patients with NHL receiving rituximab for the first time and focusing on follicular lymphoma (FL) and diffuse large B-cell lymphoma (DLBCL), which are disease types with large numbers of cases. In the process, we elucidated the importance of exploring risk factors for IRR in FL, and focused on FL cases.

Moreover, this study focused not only on patient background information and clinical test values, but also on cytokine concentrations in the blood of individual patients. The mechanism of IRR expression is believed to differ from that of immunoglobulin E-mediated type I allergies. Rituximab administration increases the levels of cytokines such as tumor necrosis factor (TNF)-α and interleukin (IL)-6 in the blood [12]. The possibility that the immunological background of each patient is related to the expression of IRR has also been investigated [13]. From this perspective, we initially conducted an analysis considering the differences between FL and DLBCL, and then focused on FL.

As described above, this study aimed to explore factors involved in the expression of IRR based on patient background information, clinical test values, and blood cytokine levels, while focusing on FL among NHL patients.

## Methods

### Patients

**Fig. 1:**
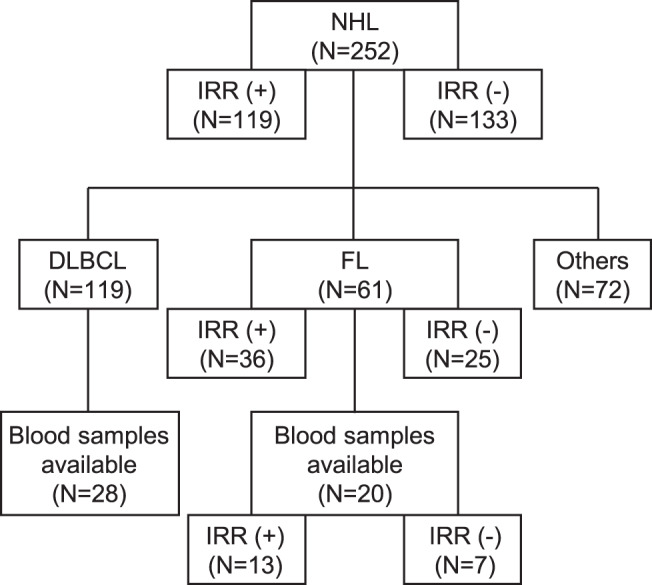
Flow diagram to illustrate patient selection methods in this study. Table 1 showed the data of IRR (+) vs IRR (-) in NHL. Figure 2 showed the data of DLBCL vs FL. Table 2-4 showed the data of IRR (+) vs IRR (-) in FL. Figure 3 showed the data of IRR (+) vs IRR (-) in blood samples available in FL. NHL: non-Hodgkin lymphoma, IRR: infusion-related reaction, DLBCL: diffuse large B-cell lymphoma, FL: follicular lymphoma

This was a single-center, retrospective, observational study of patients diagnosed with NHL who received rituximab (Rituxan^®^) at the Department of Hematology and Oncology in the University of Osaka Hospital. The exclusion criteria were a history of treatment with rituximab or rituximab biosimilars and participation in a clinical trial. Considering the patient background and frequency of IRR, it is important to conduct a disease-specific analysis to identify the risk factors for IRR. Immunological reactions are believed to be involved in IRR. To evaluate the immunological background, we measured cytokines/chemokines in blood samples from patients who provided written informed consent for blood collection. The blood samples were collected before rituximab administration. Figure 1 presents a flow diagram illustrating the patient selection process and the analytical procedures conducted in this study.

### Assessments

The following variables were analyzed: sociodemographic data, including age, sex, body weight, body surface area, body mass index, lymphoma type, IRR expression status, allergy history, medical history, and drug use history. Blood laboratory findings, including white blood cell (WBC), red blood cell, platelet, neutrophil, monocyte, lymphocyte, eosinophil, and basophil counts; hemoglobin and hematocrit levels; mean corpuscular volume; mean corpuscular hemoglobin level; mean corpuscular hemoglobin concentration; and WBC count percentages, were collected from the closest possible date prior to administration (within 3 weeks). Blood laboratories findings, including fibrin/fibrinogen degradation products (FDP), uric acid, serum creatinine, creatinine clearance, aspartate aminotransferase, alanine aminotransferase, total bilirubin, C-reactive protein, lactate dehydrogenase, soluble IL-2 receptor, and β2-microglobulin levels and estimated glomerular filtration rate, were gathered on the closest possible date prior to administration (within 3 months). All blood tests were performed at the Laboratory for Clinical Investigation in the University of Osaka Hospital.

### Rituximab administration

Patients were premedicated intravenously with 5 mg chlorpheniramine maleate and 100 mg hydrocortisone sodium succinate 30 min prior to the administration of rituximab. Rituximab was diluted in saline and administered at a dose of 375 mg/m^2^. The procedure for the first rituximab infusion was conventional; the infusion rate was set to 25 mg/h for the first 1 h, 50 mg/h for the next 0.5 h, 100 mg/h for the next 1 h, and 200 mg/h subsequently unless any adverse event developed. During rituximab administration, vital signs, including pulse, oxygen saturation, respiratory rate, and body temperature, were carefully monitored.

### IRR

Patient symptoms were collected from the records of medical professionals, such as doctors and nurses. IRRs were defined as adverse reactions that occurred within 24 h of drug administration, for which causal relationships could not be eliminated. IRR grades were determined according to the Common Terminology Criteria for Adverse Events, version 5.0 (grade 1: some symptoms appeared; grade 2: some kind of response was given to the symptoms that appeared; and grade 3: the responses to the symptoms that appeared occurred multiple times). Adverse reactions were recorded using the Medical Dictionary for Regulatory Activities (MedDRA, v26.0; http://www.meddra.org).

### Measurement of cytokine/chemokine levels in blood samples

Cytokine/chemokine levels in blood samples were measured using Bio-Plex Pro human cytokine 27-plex panel (TNF-α, interferon [IFN]-γ, IL-1β, IL-1ra, IL-2, IL-4, IL-5, IL-6, IL-7, IL-8, IL-9, IL-10, IL-12, IL-13, IL-15, IL-17, eotaxin, fibroblast growth factor basic, granulocyte colony-stimulating factor, granulocyte-macrophage colony-stimulating factor, IFN-γ inducible protein-10, monocyte chemoattractant protein-1, macrophage inflammatory protein [MIP]-1α, MIP-1β, platelet-derived growth factor-bb, regulated on activation normal T cell expressed and secreted, and vascular endothelial growth factor [VEGF]) and Bio-Plex single plex set (chemokine ligand [CCL]21, chemokine (C-X-C motif) ligand [CXCL]13, CCL24, CCL26, CX3CL1, CXCL1, CXCL9, CCL19, CXCL12, and CCL17) (Bio-Rad Laboratories, Hercules, California) according to the recommended procedures.

### Statistical analysis

The JMP^®^ Pro 15.1.0 software (SAS Institute Inc., Cary, North Carolina) was used for statistical analyses. In the univariate analysis, the chi-square analysis and Fisher’s exact test were used for binary variables, and the Wilcoxon rank sum test was used for continuous variables. Multivariate analysis was performed using logistic regression and Wald test. In all analytical tests, the criterion for significant difference was *p* < 0.05.

## Results

### IRR occurrence

**Fig. 2:**
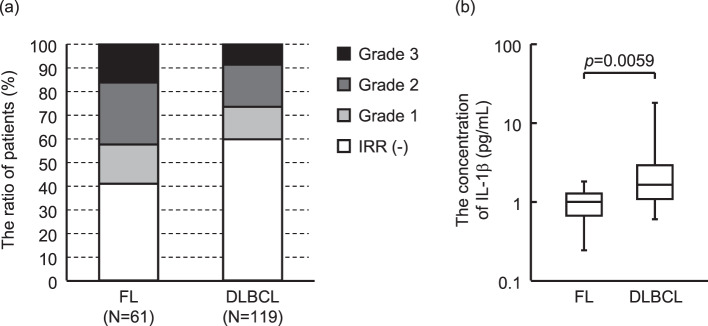
Comparison between FL and DLBCL. (**a**) The frequency and severity of IRR by FL and DLBCL. (**b**) Cytokines/chemokines in the blood samples before rituximab administration are measured using the Bio-plex system. Of the 37 cytokines/chemokines measured, the results for IL-1β, which showed a significant difference, are shown. The bottom and top box plots represent the first and third quartiles, respectively. The median is indicated by the solid line inside the box. Horizontal lines below and above the boxes indicate the lowest and highest values, respectively. Data are analyzed using the Wilcoxon rank-sum test. FL, follicular lymphoma; DLBCL, diffuse large B-cell lymphoma; IL, interleukin

In this study, IRR occurred in 119 of 252 patients (47.2%). Comparing the patient backgrounds of the IRR-expressing and non-expressing groups, significant differences were observed in lymphoma (*p* = 0.0072) and histological subtypes (*p* = 0.0081) (Table 1). Therefore, the occurrence of IRR was investigated by limiting the number of cases to those with a definitive diagnosis of FL or DLBCL. The frequency of IRR was 36/61 (59.0%) FL cases and 48/119 (40.3%) DLBCL cases, indicating that the incidence of IRR was significantly higher in FL than in DLBCL (*p* = 0.0174, Fig. 2a). In FL, the IRR severity was grade 1 in 10 patients (16.4%), grade 2 in 16 (26.2%), and grade 3 in 10 (16.4%). In DLBCL, the IRR severity was grade 1 in 16 patients (13.4%), grade 2 in 22 (18.5%), and grade 3 in 10 (8.4%). No patients with grade 4 or 5 IRR were identified in both FL and DLBCL. Comparative analysis of FL and DLBCL showed that IL-1β concentrations in FL were significantly lower than those in DLBCL (*p* = 0.0059) (Fig. 2b). The characteristics of DLBCL patients with available blood samples are shown in supplementary Table 1.

**Table 1:** The baseline characteristics of patients with IRRs and those without IRRs

Patients		IRR		
	All *n* = 252	- *n* = 133	+ *n* = 119	*p*-value
Age, mean ± SD	64.0 ± 12.5	65.8 ± 11.2	62.0 ± 13.6	0.0196^a)^
Sex, n (%)				0.3094^b)^
Male	125 (49.6)	70 (52.6)	55 (46.2)	
Female	127 (50.4)	63 (47.4)	64 (53.8)	
Rituximab dose, mean ± SD, mg	612.9 ± 75.5	617.5 ± 71.7	607.8 ± 79.5	0.3376^a)^
Lymphoma subtype, n (%)				0.0072^c)^
DLBCL	119 (47.2)	71 (53.4)	48 (40.3)	
FL	61 (24.2)	25 (18.8)	36 (30.3)	
MALT	23 (9.13)	13 (9.77)	10 (8.40)	
LPL	12 (4.76)	1 (0.75)	11 (9.24)	
MCL	8 (3.17)	5 (3.76)	3 (2.52)	
MZL	8 (3.17)	4 (3.01)	4 (3.36)	
BL	1 (0.40)	1 (0.75)	0 (0.00)	
Others	20 (7.94)	13 (9.77)	7 (5.88)	
Histological subtype, n (%)				0.0081^b)^
Indolent	118 (49.4)	51 (41.1)	67 (58.3)	
Aggressive	121 (50.6)	73 (58.9)	48 (41.7)	
Ann Arbor stage, n (%)				0.6145^b)^
I or II	53 (33.5)	29 (35.4)	24 (31.6)	
III or IV	105 (66.5)	53 (64.6)	52 (68.4)	
Splenomegaly				0.4350^b)^
Yes	42 (17.3)	20 (15.5)	22 (19.3)	
No	201 (82.7)	109 (84.5)	92 (80.7)	
Bone marrow involvement				0.0016^b)^
Yes	53 (25.6)	18 (16.5)	35 (35.7)	
No	154 (74.4)	91 (83.5)	63 (64.3)	
Chemotherapy regimen, n (%)				0.5701^b)^
Rituximab alone	99 (39.3)	48 (36.1)	51 (42.9)	
R-CHOP	77 (30.6)	45 (33.8)	32 (26.9)	
R-THPCOP	29 (11.5)	16 (12.0)	13 (10.9)	
R-B	15 (5.95)	6 (4.51)	9 (7.56)	
Others	32 (12.7)	18 (13.5)	14 (11.8)	
History of allergy				0.6579^b)^
Yes	117 (46.4)	60 (45.1)	57 (47.9)	
No	135 (53.6)	73 (54.9)	62 (52.1)	
Pollinosis, n (%)				0.4585^b)^
Yes	45 (17.9)	26 (19.6)	19 (16.0)	
No	207 (82.1)	107 (80.5)	100 (84.0)	
Drug allergy, n (%)				0.2187^b)^
Yes	53 (21.0)	24 (18.1)	29 (24.4)	
No	199 (79.0)	109 (82.0)	90 (75.6)	
Food allergy, n (%)				0.2793^b)^
Yes	36 (14.3)	22 (16.5)	14 (11.8)	
No	216 (85.7)	111 (83.5)	105 (88.2)	
Asthma				0.0163^b)^
Yes	19 (7.54)	5 (3.76)	14 (11.8)	
No	233 (92.5)	128 (96.2)	105 (88.2)	
Cardiovascular disease, n (%)				0.9440^b)^
Yes	118 (46.8)	62 (46.6)	56 (47.1)	
No	134 (53.2)	71 (53.4)	63 (52.9)	
Pulmonary disease, n (%)				0.0934^b)^
Yes	74 (29.4)	33 (24.8)	41 (34.5)	
No	178 (70.6)	100 (75.2)	78 (65.6)	

**R-CHOP**: rituximab, cyclophosphamide, doxorubicin, vincristine, prednisolone; R-THPCOP: rituximab, pirarubicin, cyclophosphamide, vincristine, prednisolone; R-B: rituximab, bendamustine. DLBCL: diffuse large B-cell lymphoma; FL: follicular lymphoma; MALT: mucosa associated lymphoid tissue; LPL: lymphoplasmacytic lymphoma; ML: Mantle lymphoma; MZL: Marginal zone lymphoma; BL: Burkitt lymphoma. Data was analyzed with (a) Wilcoxon rank sum test, (b) Pearson’s chi-square test, (c) Fisher’s exact test

### Risk factors of IRR in patients with FL

Ann Arbor stage III or IV (*p* = 0.0349) and drug allergies (*p* = 0.0344) were significantly associated with IRR expression (Table 2). Analysis of blood test values showed that the incidence of IRR was significantly higher in patients with a low neutrophil ratio (*p* = 0.0006), high lymphocyte ratio (*p* = 0.0012), or high lymphocyte number (*p* = 0.0165) (Table 3). Since these three blood test values are thought to be related, we focused on the lymphocyte count. A receiver operating characteristic curve was created to confirm the cutoff value, which was 1530 cells/μL. Thereafter, we compared the < 1,600 and ≥ 1600 cells/μL groups (*p* = 0.0003). Since this study focused on patients with FL, the number of cases was small, and the number of factors selected for multivariate analysis had to be limited to two. Of the three factors of the Ann Arbor stage, drug allergies, and lymphocyte count, a multivariate analysis was performed based on the lymphocyte count and Ann Arbor stage (including extranodal lesions, bone marrow involvement, and splenomegaly, which affect classification), which have been frequently reported as risk factors for IRR in previous studies (Table 4) (1–5, 8–10). This elucidated that only the lymphocyte count was significantly associated (*p* = 0.0162).

**Table 2:** Comparison of patient’s characteristics of FL patients with IRRs and without IRRs

Patients		IRR		
	All *n* = 61	- *n* = 25	+ *n* = 36	*p*-value
Age, mean ± SD	64.1 ± 8.75	66.6 ± 7.62	62.4 ± 9.18	0.0756^a)^
Sex, n (%)				0.0918^b)^
Male	24 (39.3)	13 (52.0)	11 (30.6)	
Female	37 (60.7)	12 (48.0)	25 (69.4)	
Body Weight, mean ± SD, kg	58.2 ± 11.0	58.3 ± 11.2	58.2 ± 11.0	0.8661^a)^
Body Mass Index, mean ± SD	22.5 ± 3.32	22.1 ± 3.14	22.7 ± 3.47	0.6867^a)^
Body surface area, mean ± SD, m^2^	1.60 ± 0.17	1.61 ± 0.18	1.59 ± 0.17	0.7749^a)^
Rituximab dose, mean ± SD, mg	612 ± 70.6	618 ± 68.8	608 ± 72.4	0.5986^a)^
Ann Arbor stage, n (%)				0.0349^c)^
I or II	6 (12.2)	5 (25.0)	1 (3.45)	
III or IV	43 (87.8)	15 (75.0)	28 (96.6)	
Splenomegaly				0.1434^b)^
Yes	13 (22.0)	3 (12.5)	10 (28.6)	
No	46 (78.0)	21 (87.5)	25 (71.4)	
Bone marrow involvement				0.0766^b)^
Yes	24 (43.6)	6 (28.6)	18 (52.9)	
No	31 (56.4)	15 (71.4)	16 (47.1)	
Chemotherapy regimen, n (%)				0.7146^c)^
Rituximab alone	30 (49.2)	14 (56.0)	16 (44.4)	
R-CHOP	15 (24.6)	7 (28.0)	8 (22.2)	
R-THPCOP	3 (4.92)	1 (4.00)	2 (5.56)	
R-B	9 (14.8)	2 (8.00)	7 (19.4)	
Others	4 (6.56)	1 (4.00)	3 (8.33)	
History of allergy				0.2703^b)^
Yes	32 (52.5)	11 (44.0)	21 (58.3)	
No	29 (47.5)	14 (56.0)	15 (41.7)	
Pollinosis, n (%)				0.2541^c)^
Yes	8 (13.1)	5 (20.0)	3 (8.33)	
No	53 (86.9)	20 (80.0)	33 (91.7)	
Drug allergy, n (%)				0.0344^b)^
Yes	13 (21.3)	2 (8.00)	11 (30.6)	
No	48 (78.7)	23 (92.0)	25 (69.4)	
Food allergy, n (%)				1.0000^c)^
Yes	9 (14.8)	4 (16.0)	5 (13.9)	
No	52 (85.3)	21 (84.0)	31 (86.1)	
Asthma				0.1371^c)^
Yes	4 (6.56)	0 (0.00)	4 (11.1)	
No	57 (93.4)	25 (100)	32 (88.9)	
Cardiovascular disease, n (%)				0.5765^b)^
Yes	34 (55.7)	15 (60.0)	19 (52.8)	
No	27 (44.3)	10 (40.0)	27 (47.2)	
Pulmonary disease, n (%)				0.3277^c)^
Yes	12 (19.7)	3 (12.0)	9 (25.0)	
No	49 (80.3)	22 (88.0)	27 (75.0)	
Drug use history from the day before administration				
Antipyretics, n (%)				0.5624^c)^
Yes	3 (4.9)	2 (8.0)	1 (2.8)	
No	58 (95.1)	23 (92.0)	35 (97.2)	
Antiallergic drugs, n (%)				1.0000^c)^
Yes	4 (6.56)	2 (8.0)	2 (5.6)	
No	57 (93.4)	23 (92.0)	34 (94.4)	
Steroids, n (%)				0.3924^c)^
Yes	5 (8.2)	3 (12.0)	2 (5.6)	
No	56 (91.8)	22 (88.0)	34 (94.4)	

**R-CHOP**: rituximab, cyclophosphamide, doxorubicin, vincristine, prednisolone; R-THPCOP: rituximab, pirarubicin, cyclophosphamide, vincristine, prednisolone; R-B: rituximab, bendamustine, FL: follicular lymphoma. Data was analyzed with (a) Wilcoxon rank sum test, (b) Pearson’s chi-square test, (c) Fisher’s exact test

**Table 3:** Comparisons of laboratory values of FL patients with IRRs and without IRRs

			IRR		
		All *n* = 61	- *n* = 25	+ *n* = 36	*p*-value
WBC, 10^3^/µL		6.56 ± 3.26	6.27 ± 2.50	6.76 ± 3.72	0.9357
RBC, 10^6^/µL		4.34 ± 0.54	4.42 ± 0.59	4.27 ± 0.50	0.4906
Hemoglobin, g/dL		13.0 ± 1.49	13.3 ± 1.50	12.8 ± 1.46	0.2647
Hematocrits, %		39.3 ± 4.16	40.1 ± 4.19	38.8 ± 4.12	0.4859
Platelets, 10^3^/µL		220 ± 55.5	228 ± 52.7	215 ± 57.5	0.4199
Neutrophils	Ratio, %	65.0 ± 13.3	71.7 ± 7.53	60.4 ± 14.5	0.0006
	Number, 10^3^/µL	4.16 ± 1.95	4.57 ± 2.08	3.87 ± 1.83	0.1132
Lymphocytes	Ratio, %	25.5 ± 13.8	18.8 ± 6.59	30.1 ± 15.9	0.0012
	Number, 10^3^/µL	1.81 ± 2.01	1.09 ± 0.38	2.30 ± 2.50	0.0165
Monocytes	Ratio, %	6.32 ± 2.49	6.52 ± 1.57	6.19 ± 2.99	0.8718
	Number, 10^3^/µL	0.40 ± 0.29	0.40 ± 0.17	0.40 ± 0.35	0.5723
Eosinophils	Ratio, %	2.50 ± 2.20	2.38 ± 2.27	2.59 ± 2.17	0.7083
	Number,/µL	155.7 ± 200.9	163.5 ± 284.3	150.2 ± 116.8	0.4414
Basophils	Ratio, %	0.66 ± 0.47	0.63 ± 0.35	0.68 ± 0.42	0.7405
	Number,/µL	39.9 ± 24.6	38.1 ± 24.1	41.1 ± 25.2	0.5428
FDP, µg/mL		0.96 ± 0.99	0.83 ± 0.60	1.05 ± 1.19	0.7871
Uric acid, mg/dL		5.18 ± 1.58	5.37 ± 1.88	5.05 ± 1.34	0.2289
Serum creatinine, mg/dL		0.73 ± 0.17	0.77 ± 0.16	0.70 ± 0.17	0.0676
eGFR, mL/(min・1.73 m^2^)		72.4 ± 12.8	69.5 ± 10.8	74.5 ± 13.8	0.0990
Creatinine clearance, mL/min		41.9 ± 17.5	43.9 ± 17.7	40.4 ± 17.5	0.3442
AST, U/L		22.6 ± 8.20	22.4 ± 9.22	22.7 ± 7.55	0.4705
ALT, U/L		19.0 ± 14.0	18.2 ± 14.7	19.5 ± 13.7	0.7132
LDH, U/L		203 ± 61.2	218 ± 60.1	193 ± 60.4	0.0689
Total bilirubin, mg/dL		0.57 ± 0.26	0.58 ± 0.20	0.56 ± 0.29	0.4301
C-reactive protein, mg/dL		0.23 ± 0.38	0.20 ± 0.38	0.25 ± 0.39	0.4000
sIL-2 R, U/mL		1496 ± 1919	1299 ± 2147	1632 ± 1763	0.1405
β2-microglobulin, μg/mL		2.30 ± 1.00	2.34 ± 1.12	2.27 ± 0.91	0.8979

WBC: white blood cell; RBC; red blood cell; FDP: fibrin degradation products; eGFR: estimated glomerular filtration rate; AST: aspartate transferase; ALT: alanine transaminase; LDH: lactate dehydrogenase; sIL-2 R: soluble interleukin-2 receptor. Data presented as mean ± SD unless otherwise indicated. Data was analyzed with Wilcoxon rank sum test

**Table 4:** Multivariate logistic regression analysis for presence of IRR in FL

Variable	OR	95% CI	*p***-value**
Ann Arbor stage			
III or IV vs I or II	5.00	0.520–48.1	0.1634
Number of lymphocytes (/µL)			
≥1,600 vs < 1,600	14.0	1.63–120	0.0162

OR: Odds Ratio, CI: Confidence Interval. Statistical significance was evaluated by logistic regression analysis and Wald test

### Association between the cytokines/chemokine levels and IRR in patients with FL

**Fig. 3:**
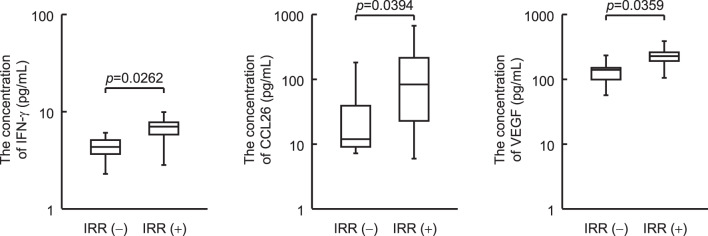
Comparison of cytokine/chemokine between patients with FL with IRRs and without IRRs. Cytokines/chemokines in the blood samples before rituximab administration are measured using the Bio-plex system. Of the 37 cytokines/chemokines measured, IFN-γ, CCL26, and VEGF show significant differences. The bottom and top box plots represent the first and third quartiles, respectively. The median is indicated by the solid line inside the box. Horizontal lines below and above the boxes indicate the lowest and highest values, respectively. Data are analyzed using the Wilcoxon rank-sum test. IFN, interferon; CCL, chemokine ligand; VEGF, vascular endothelial growth factor; FL, follicular lymphoma; IRRs, infusion-related reactions

Univariate analysis of cytokine/chemokine concentrations was performed for only 20 patients with FL using blood samples, and the results showed that high levels of IFN-γ (*p* = 0.0262), CCL26 (*p* = 0.0394), and VEGF (*p* = 0.0359) were significantly associated with IRR (Fig. 3). The baseline characteristics and laboratory values of these 20 patients are presented in supplementary tables 2 and 3. Although the number of cases was small and the statistical analysis is only for reference, no significant differences in patient’s characteristics were observed regarding the expression of IRR. Significant differences were observed in neutrophil ratio, and cases with IRR tended to have higher lymphocyte ratio and lymphocyte number; this result was consistent with that obtained by analysis of all FL patients.

## Discussion

This study showed that the incidence of IRR differed significantly between patients with FL and those with DLBCL. In the treatment of FL, an indolent lymphoma, follow-up observations are often performed until the appearance of symptoms. A previous study reported that IRRs are likely to occur because the tumor burden has increased at the time of rituximab administration [1]. This suggests that it is important to narrow the analysis to a specific disease when studying factors related to IRR expression.

In addition, a comparative analysis of FL and DLBCL by measuring cytokine/chemokine levels showed that IL-1β levels in FL were significantly lower than those in DLBCL. A previous study has also shown that IL-1β levels differ between patients with FL and those with DLBCL, but the IL-1β levels were higher in patients with FL [14], which is the opposite of the results of the present study. The previous study included 36 and 55 cases of FL and DLBCL, respectively, and the number of cases was limited, as in our study. The method used allows for simultaneous measurement of multiple cytokines and chemokines in a small sample volume, which is slightly less sensitive and accurate than methods measuring each cytokine and chemokine individually. The cytokines and chemokines measured were summarized in supplementary Fig. 4. As with a previous study, there was individual variability in their measurements. Further analysis of additional cases and use of more sensitive methods are warranted. However, the results showed that the baselines differed between diseases, and it is important to separate diseases when examining the relationship between the IRR and cytokines, chemokines, and other factors.

IRR in FL is associated with the Ann Arbor stage and lymphocyte count. These factors are thought to be related to the amount of tumor, which is consistent with previous reports that a large amount of tumor is associated with the incidence of IRR [8, 15]. Previous reports have shown that lymphocyte counts of 3,500/µL or 5,000/µL or more are associated with the incidence of IRR [2, 16], and this study also confirmed that lymphocyte count is an important risk factor for IRR development. The multivariate analysis revealed no significant difference in the Ann Arbor stage. In contrast, lymphocyte count was found to be a significant predictor of IRR, suggesting that it is a direct, quantitative predictor of IRR. The results of this study indicate that a relatively low lymphocyte count of ≥1,600/µL is a predictor of IRR incidence. This suggests the importance of limiting the target disease to FL and verifying the risk factors for the occurrence of IRR. In addition, a significant association was confirmed between drug allergies and IRR. Previous reports have shown that the IRR incidence is significantly higher in cases of drug allergy [5] or in cases with unknown allergy registration [17]. Due to the small number of cases in this study, it was necessary to limit the multivariate analysis to two factors, and it was not possible to evaluate factors including allergies. Further verification using an increased number of cases is necessary. This indicated that the patient’s immunological predisposition may be related to IRR expression, and it is expected that comprehensive verification of this with the results of cytokine/chemokine measurements will lead to the elucidation of the risk factors for IRR expression and even the mechanism of IRR expression.

The use of steroids, antiallergic drugs, and antipyretics prior to rituximab administration may influence the expression of IRR. In this study, we focused on FL, while there were few cases using drugs that are thought to affect IRR expression, and no association with IRR onset was observed (Table 2). In contrast, there are many examples of combined therapy with cytotoxic anticancer drugs and prednisolone in DLBCL, and analysis taking into account their effects is necessary, and more detailed verification is under process.

The infusion rate set at Osaka University Hospital is slower than that indicated in the package insert. To prevent IRR, it is recommended to start with a slow infusion rate and gradually increase it. For safety reasons, our hospital has decided to start with an infusion rate even slower than that recommended. As this study was conducted at a single center, we think that the infusion rate will have little impact on the analysis of risk factors for IRR.

Univariate analysis of cytokines/chemokines was performed on only the 20 patients with FL from whom blood samples were obtained, and the IFN-γ, VEGF, and CCL26 levels were significantly associated with IRR expression. IFN-γ is a proinflammatory cytokine that activates macrophages and natural killer (NK) cells. Since macrophages and NK cells exhibit ADCC, it is speculated that high IFN-γ levels before rituximab administration would lead to a stronger ADCC effect of rituximab and greater release of cytokines. In addition, in a report of chronic lymphocytic leukemia cases treated with obinutuzumab, the number of patients in the IRR expression group was significantly higher than that in the non-expression group, as in the present study [13]. Therefore, it is suggested that IFN-γ is also useful for predicting IRR expression in patients with FL treated with rituximab. In addition, because CCL26 is a chemokine that induces the migration of eosinophils and basophils, the migrated eosinophils and basophils may cause IRR, which has symptoms similar to an allergic reaction. Furthermore, VEGF is upregulated in tumor cells and involved in the invasion of solid tumors through cell proliferation and migration via angiogenesis [18]. High serum VEGF levels are associated with decreased progression-free survival, and high microvessel density in the interfollicular regions of FL is associated with disease progression and decreased overall survival, suggesting that VEGF-induced angiogenesis is involved in the pathology of FL [19]. Therefore, even in FL, which is a type of lymphoma rather than a solid tumor, it is plausible that there might be a relationship between VEGF and tumor mass due to VEGF-induced angiogenesis, and that there might also be a relationship with IRR expression. VEGF is involved not only in angiogenesis but also in increased vascular permeability, which affects tumor cell metastasis [20]. Therefore, it is speculated that the increased vascular permeability induced by VEGF facilitates the induction of inflammatory responses, and it is possible that such factors lead to IRR expression owing to high VEGF levels.

Regarding the patient background and clinical test values of the 20 FL cases from which blood samples were obtained, the same trends in neutrophil/lymphocyte counts were observed as in all FL cases. The highest correlation coefficient between IFN-γ, CCL26, or VEGF and neutrophil or lymphocyte counts was 0.5366 for lymphocyte count and IFN-γ, which indicated no high correlation. Our results demonstrated that lymphocyte count was useful for predicting the onset of IRR in all FL cases. However, for more sensitive predictions, we believe that analysis including cytokines, rather than lymphocyte count alone, is important. Unfortunately, the case number was small, thereby preventing multivariate analysis including blood cytokine levels. Further expansion of the study, such as by adding additional cases, is required. Additionally, we plan to conduct a prospective study in which blood samples are collected over time before and after rituximab administration to evaluate the blood levels of cytokines/chemokines and contribute to the development of a method to more accurately predict the onset of IRR. In addition, the mechanism of IRR onset remains unclear, and we hope to elucidate this through these studies.

The limitations of this study include the limited information that could be obtained because it was a retrospective study, bias in IRR assessment or data collection because IRR expression was based on the subjective assessment of doctors or nurses, the fact that it was a single-center study, and the small number of cases, which means that sufficient consideration could not be given to this study. Despite the scope for further investigation is needed, this study highlights the importance of limiting the target diseases.

## Conclusions

The incidence of IRR differed significantly between patients with FL and those with DLBCL. In FL, measuring lymphocyte count can help predict the IRR occurrence; furthermore, it was suggested that measuring cytokine and chemokine levels in the blood before rituximab treatment may be able to more effectively predict the IRR occurrence in the future. Further studies like this research may enable more accurate prediction and avoidance of IRR, leading to the provision of safe and useful treatment.

## Electronic supplementary material

Below is the link to the electronic supplementary material.

Supplementary Material 1

## Data Availability

The datasets used and analyzed during the current study are available from the corresponding author on reasonable request.

## Abbreviations

ADCC: Antibody-dependent cellular cytotoxicity
CCL: Chemokine ligand
CXCL: Chemokine (C-X-C motif) ligand
DLBCL: Diffuse large B-cell lymphoma
FDP: Fibrin/fibrinogen degradation products
FL: Follicular lymphoma
IFN: Interferon
IL: Interleukin
IRR: Infusion-related reaction
MIP: Macrophage inflammatory protein
NK: Natural killer
NHL: Non-Hodgkin lymphoma
TNF: Tumor necrosis factor
VEGF: Vascular endothelial growth factor
WBC: White blood cell
