# Supplementary material for: Investigating rituximab-induced infusion-related reactions in patients with non-Hodgkin lymphoma, with a focus on follicular lymphoma: a retrospective observational study
Source: J Pharm Health Care Sci. 2026 Jan 12;12:17. doi: 10.1186/s40780-026-00538-6 (PMC12888495; doi:10.1186/s40780-026-00538-6)
Supplement: Supplementary file 1 — Supplementary Material 1 [file 40780_2026_538_MOESM1_ESM.docx]

Supplementary table 1 Patient’s characteristics of DLBCL patients with available blood samples

| Patients |  |
| --- | --- |
| Age, mean ± SD | 67.6 ± 8.52 |
| Sex, n (%) |  |
| Male | 12 (42.9) |
| Female | 16 (57.1) |
| Ann Arbor stage, n (%) |  |
| I or II | 11 (52.4) |
| III or IV | 10 (47.6) |
| Splenomegaly |  |
| Yes | 3 (10.7) |
| No | 25 (89.3) |
| Bone marrow involvement |  |
| Yes | 1 (4.2) |
| No | 23 (95.8) |
| Chemotherapy regimen, n (%) |  |
| Rituximab alone | 2 (7.1) |
| R-CHOP | 15 (53.6) |
| R-THPCOP | 11 (39.3) |
| History of allergy |  |
| Yes | 16 (57.1) |
| No | 12 (42.9) |
| Pollinosis, n (%) |  |
| Yes | 4 (14.3) |
| No | 24 (85.7) |
| Drug allergy, n (%) |  |
| Yes | 9 (32.1) |
| No | 19 (67.9) |
| Food allergy, n (%) |  |
| Yes | 5 (17.9) |
| No | 23 (82.1) |

R-CHOP: rituximab, cyclophosphamide, doxorubicin, vincristine, prednisolone; R-THPCOP: rituximab, pirarubicin, cyclophosphamide, vincristine, prednisolone; DLBCL: diffuse large B-cell lymphoma.

Supplementary table 2 Comparison of patient’s characteristics between FL patients with and without IRR for whom blood samples were available

| Patients |  | IRR | |  |
| --- | --- | --- | --- | --- |
|  | All  n = 20 | -  n = 7 | +  n = 13 | *p*-value |
| Age, mean ± SD | 65.6 ± 8.64 | 69.7 ± 8.35 | 63.4 ± 8.25 | 0.1538^a)^ |
| Sex, n (%) |  |  |  | 0.3285^b)^ |
| Male | 7 (35.0) | 1 (14.3) | 6 (46.2) |  |
| Female | 13 (65.0) | 6 (85.7) | 7 (53.8) |  |
| Ann Arbor stage, n (%) |  |  |  |  |
| I or II | 0 (0.0) | 0 (0.0) | 0 (0.0) |  |
| III or IV | 17 (100.0) | 6 (100.0) | 11 (100.0) |  |
| Splenomegaly |  |  |  | 1.0000^b)^ |
| Yes | 7 (35.0) | 2 (28.6) | 5 (38.5) |  |
| No | 13 (65.0) | 5 (71.4) | 8 (61.5) |  |
| Bone marrow involvement |  |  |  | 0.1577^b)^ |
| Yes | 8 (40.0) | 1 (14.3) | 7 (53.8) |  |
| No | 12 (60.0) | 6 (85.7) | 6 (46.2) |  |
| Chemotherapy regimen, n (%) |  |  |  | 0.2105^b)^ |
| Rituximab alone | 6 (30.0) | 1 (14.3) | 5 (38.5) |  |
| R-CHOP | 6 (30.0) | 4 (57.1) | 2 (15.4) |  |
| R-THPCOP | 2 (10.0) | 1 (14.3) | 1 (7.7) |  |
| R-B | 6 (30.0) | 1 (14.3) | 5 (15.4) |  |
| History of allergy |  |  |  | 1.0000^b)^ |
| Yes | 11 (55.0) | 4 (57.1) | 7 (53.8) |  |
| No | 9 (45.0) | 3 (42.9) | 6 (46.2) |  |
| Pollinosis, n (%) |  |  |  | 0.5868^b)^ |
| Yes | 4 (20.0) | 2 (28.6) | 2 (15.4) |  |
| No | 16 (80.0) | 5 (71.4) | 11 (84.6) |  |
| Drug allergy, n (%) |  |  |  | 0.3285^b)^ |
| Yes | 7 (35.0) | 1 (14.3) | 6 (46.2) |  |
| No | 13 (65.0) | 6 (85.7) | 7 (53.8) |  |
| Food allergy, n (%) |  |  |  | 0.5868^b)^ |
| Yes | 4 (20.0) | 2 (28.6) | 2 (15.4) |  |
| No | 16 (80.0) | 5 (71.4) | 11 (84.6) |  |

R-CHOP: rituximab, cyclophosphamide, doxorubicin, vincristine, prednisolone; R-THPCOP: rituximab, pirarubicin, cyclophosphamide, vincristine, prednisolone; R-B: rituximab, bendamustine, FL: follicular lymphoma. Data was analyzed with (a) Wilcoxon rank sum test, (b) Fisher's exact test

Supplementary table 3 Comparison of laboratory values between FL patients with and without IRR for whom blood samples were available

|  | |  | IRR | |  |
| --- | --- | --- | --- | --- | --- |
|  | | All  n = 20 | -  n = 7 | +  n = 13 | *p*-value |
| WBC, 10^3^/µL | | 6.23 ± 3.36 | 5.85 ± 1.35 | 6.44 ± 4.10 | 0.4757 |
| RBC, 10^6^/µL | | 4.35 ± 0.49 | 4.36 ± 0.47 | 4.35 ± 0.52 | 0.6919 |
| Hemoglobin, g/dL | | 13.1 ± 1.63 | 13.3 ± 1.34 | 13.0 ± 1.81 | 0.9684 |
| Hematocrits, % | | 39.7 ± 4.39 | 40.0 ± 3.69 | 39.5 ± 4.87 | 0.8741 |
| Platelets, 10^3^/µL | | 216 ± 51.7 | 223 ± 47.1 | 211 ± 55.3 | 0.6345 |
| Neutrophils | Ratio, % | 65.7 ± 11.7 | 74.6 ± 9.0 | 60.9 ± 10.3 | 0.0174 |
|  | Number, 10^3^/µL | 4.06 ± 1.94 | 4.45 ± 1.55 | 3.84 ± 2.15 | 0.2346 |
| Lymphocytes | Ratio, % | 24.0 ± 11.6 | 16.5 ± 7.6 | 27.9 ± 11.7 | 0.0521 |
|  | Number, 10^3^/µL | 1.53 ± 1.45 | 0.89 ± 0.28 | 1.87 ± 1.70 | 0.0684 |
| Monocytes | Ratio, % | 6.78 ± 2.94 | 6.21 ± 1.71 | 7.08 ± 3.45 | 0.6626 |
|  | Number, 10^3^/µL | 0.45 ± 0.44 | 0.36 ± 0.11 | 0.50 ± 0.55 | 0.8741 |
| Eosinophils | Ratio, % | 2.78 ± 2.42 | 2.00 ± 0.89 | 3.20 ± 2.89 | 0.6628 |
|  | Number, /µL | 154.5 ± 127.0 | 113.4 ± 57.3 | 176.6 ± 149.7 | 0.5791 |
| Basophils | Ratio, % | 0.80 ± 0.41 | 0.66 ± 0.34 | 0.87 ± 0.44 | 0.2333 |
|  | Number, /µL | 45.1 ± 25.7 | 36.7 ± 17.6 | 49.7 ± 28.8 | 0.3834 |
| FDP, µg/mL | | 1.14 ± 1.37 | 0.98 ± 0.66 | 1.21 ± 1.62 | 0.7588 |
| Uric acid, mg/dL | | 5.40 ± 1.41 | 5.14 ± 1.59 | 5.53 ± 1.36 | 0.6628 |
| Serum creatinine, mg/dL | | 0.73 ± 0.19 | 0.66 ± 0.13 | 0.76 ± 0.21 | 0.3823 |
| eGFR, mL/(min・1.73m^2^) | | 71.4 ± 12.7 | 71.1 ± 8.1 | 71.6 ± 14.9 | 0.5261 |
| Creatinine clearance, mL/min | | 79.1 ± 23.1 | 68.0 ± 16.0 | 85.2 ± 24.6 | 0.0961 |
| AST, U/L | | 23.9 ± 8.03 | 24.1 ± 7.27 | 23.7 ± 8.70 | 0.7807 |
| ALT, U/L | | 20.4 ± 15.3 | 15.4 ± 6.58 | 23.0 ± 18.1 | 0.4265 |
| LDH, U/L | | 214 ± 77.7 | 244 ± 74.3 | 198 ± 77.4 | 0.1909 |
| Total bilirubin, mg/dL | | 0.65 ± 0.37 | 0.67 ± 0.23 | 0.64 ± 0.43 | 0.4469 |
| C-reactive protein, mg/dL | | 0.43 ± 0.58 | 0.46 ± 0.66 | 0.41 ± 0.56 | 0.9363 |
| sIL-2R, U/mL | | 1408 ± 1505 | 830 ± 304 | 1719 ± 1800 | 0.5791 |
| β2-microglobulin, μg/mL | | 2.34 ± 0.87 | 2.10 ± 0.22 | 2.46 ± 1.06 | 0.7801 |

WBC: white blood cell; RBC; red blood cell; FDP: fibrin degradation products; eGFR: estimated glomerular filtration rate; AST: aspartate transferase; ALT: alanine transaminase; LDH: lactate dehydrogenase; sIL-2R: soluble interleukin-2 receptor; FL: follicular lymphoma. Data presented as mean ± SD unless otherwise indicated. Data was analyzed with Wilcoxon rank sum test.

Supplementary table 4 Comparison of laboratory values between FL patients with and without IRR for whom blood samples were available

|  | FL | | |  | DLBCL |
| --- | --- | --- | --- | --- | --- |
|  |  | IRR | |  |  |
|  | All  n = 20 | -  n = 7 | +  n = 13 |  | All  n =28 |
| TNF-α (pg/mL) | 89.7 ± 61.4 | 68.2 ± 22.6 | 101 ± 72.8 |  | 125 ± 121 |
| IFN-γ (pg/mL) | 5.95 ± 2.12 | 4.38 ± 1.34 | 6.89 ± 1.97 |  | 8.49 ± 8.49 |
| IL-1β (pg/mL) | 1.03 ± 0.46 | 0.95 ± 0.42 | 1.07 ± 0.49 |  | 2.91 ± 4.00 |
| IL-1ra (pg/mL) | 571 ± 282 | 510 ± 269 | 607 ± 294 |  | 804 ± 605 |
| IL-2 (pg/mL) | 9.80 ± 3.45 | 8.93 ± 3.33 | 10.1 ± 3.81 |  | 11.7 ± 6.30 |
| IL-4 (pg/mL) | 3.43 ± 1.27 | 2.95 ± 0.87 | 3.69 ± 1.41 |  | 4.21 ± 1.61 |
| IL-5 (pg/mL) | 24.8 ± 7.37 | 23.8^*^ | 25.2 ± 8.99 |  | 85.8 ± 197 |
| IL-6 (pg/mL) | 5.14 ± 3.73 | 7.38 ± 6.09 | 4.02 ± 1.32 |  | 11.5 ± 29.8 |
| IL-7 (pg/mL) | 23.2 ± 6.90 | 20.2 ± 7.66 | 24.9 ± 6.08 |  | 31.7 ± 23.6 |
| IL-8 (pg/mL) | 78.2 ± 83.2 | 98.9 ± 115 | 66.2 ± 60.4 |  | 103 ± 155 |
| IL-9 (pg/mL) | 77.2 ± 21.9 | 69.2 ± 24.6 | 81.4 ± 20.0 |  | 94.4 ± 64.2 |
| IL-10 (pg/mL) | 14.1 ± 9.10 | 10.6 ± 2.85 | 14.9 ± 9.93 |  | 61.1 ± 207 |
| IL-12 (pg/mL) | 7.38 ± 1.73 | 7.88 ± 2.12 | 6.38^*^ |  | 104 ± 171 |
| IL-13 (pg/mL) | 8.47 ± 7.25 | 6.08 ± 5.42 | 9.75 ± 7.96 |  | 11.6 ± 18.0 |
| IL-15 (pg/mL) | 261 ± 73.9 | 244 ± 74.5 | 270 ± 79.2 |  | 337 ± 256 |
| IL-17 (pg/mL) | 15.9 ± 5.41 | 14.8 ± 5.21 | 16.4 ± 5.65 |  | 20.4 ± 10.2 |
| Eotaxin (pg/mL) | 123 ± 37.5 | 111 ± 31.7 | 130 ± 39.9 |  | 140 ± 64.0 |
| Fibroblast growth factor basic (pg/mL) | 30.0 ± 6.42 | 31.8 ± 5.22 | 29.0 ± 6.98 |  | 31.8 ± 11.3 |
| Granulocyte colony-stimulating factor (pg/mL) | 360 ± 291 | 385 ± 336 | 346 ± 277 |  | 325 ± 214 |
| Granulocyte-macrophage colony-stimulating factor (pg/mL) | 3.12 ± 1.52 | 2.71 ± 1.47 | 3.20 ± 1.60 |  | 6.49 ± 14.4 |
| IFN-γ inducible protein-10 (ng/mL) | 1.09 ± 0.66 | 1.12 ± 0.78 | 1.07 ± 0.62 |  | 1.72 ± 1.59 |
| Monocyte chemoattractant protein-1 (pg/mL) | 86.7 ± 55.1 | 56.6 ± 25.8 | 103 ± 60.4 |  | 93.4 ± 53.8 |
| MIP-1α (pg/mL) | 24.3 ± 26.5 | 26.9 ± 32.1 | 22.9 ± 24.2 |  | 38.6 ± 81.5 |
| MIP-1β (pg/mL) | 191 ± 122 | 158 ± 44.6 | 209 ± 146 |  | 204 ± 140 |
| Platelet-derived growth factor-bb (ng/mL) | 5.40 ± 2.72 | 4.92 ± 2.91 | 5.66 ± 2.69 |  | 5.62 ± 2.44 |
| Regulated on activation normal T cell expressed and secreted (ng/mL) | 8.74 ± 2.13 | 8.36 ± 2.52 | 9.06 ± 1.92 |  | 10.7 ± 2.79 |
| VEGF (pg/mL) | 193 ± 84.1 | 138 ± 59.5 | 232 ± 78.7 |  | 237 ± 87.9 |
| CCL21 (ng/mL) | 162 ± 178 | 100 ± 89.9 | 195 ± 207 |  | 95.7 ± 71.7 |
| CXCL13 (ng/mL) | 1.30 ± 2.28 | 2.02 ± 3.68 | 0.91 ± 0.99 |  | 0.97 ± 1.75 |
| CCL24 (ng/mL) | 3.36 ± 3.54 | 3.51 ± 3.93 | 3.28 ± 3.48 |  | 2.75 ± 3.06 |
| CCL26 (pg/mL) | 114 ± 165 | 41.2 ± 62.7 | 154 ± 191 |  | 82.4 ± 68.7 |
| CX3CL1 (ng/mL) | 1.10 ± 0.87 | 1.13 ± 0.98 | 1.08 ± 0.85 |  | 1.26 ± 0.95 |
| CXCL1 (pg/mL) | 729 ± 287 | 777 ± 363 | 703 ± 251 |  | 797 ± 366 |
| CXCL9 (ng/mL) | 4.98 ± 6.72 | 5.79 ± 8.50 | 4.67 ± 6.29 |  | 7.82 ± 12.1 |
| CCL19 (ng/mL) | 1.81 ± 2.57 | 0.94 ± 0.52 | 2.28 ± 3.11 |  | 1.37 ± 2.23 |
| CXCL12 (ng/mL) | 16.8 ± 6.01 | 14.6 ± 3.52 | 18.0 ± 6.84 |  | 19.1 ± 7.07 |
| CCL17 (ng/mL) | 3.36 ± 3.68 | 1.78 ± 1.53 | 4.36 ± 4.33 |  | 2.16 ± 2.87 |

FL: follicular lymphoma; DLBCL: diffuse large B-cell lymphoma; TNF: tumor necrosis factor, IFN: interferon, IL: interleukin, MIP: macrophage inflammatory protein, VEGF: vascular endothelial growth factor, CCL: chemokine ligand, CXCL: chemokine (C-X-C motif) ligand. Data presented as mean ± SD unless otherwise indicated. ^*^: The SD could not be calculated because the concentration could only be calculated for one sample due to the detection limit of the calibration curve.
